# Magnetic Nanosystem for Cancer Therapy Using Oncocalyxone A, an Antitomour Secondary Metabolite Isolated from a Brazilian Plant

**DOI:** 10.3390/ijms140918269

**Published:** 2013-09-05

**Authors:** Antônio C. H. Barreto, Vivian R. Santiago, Rafael M. Freire, Selma E. Mazzetto, Juliano C. Denardin, Giuseppe Mele, Igor M. Cavalcante, Maria E. N. P. Ribeiro, Nágila M. P. S. Ricardo, Tamara Gonçalves, Luigi Carbone, Telma L. G. Lemos, Otília D. L. Pessoa, Pierre B. A. Fechine

**Affiliations:** 1 Advanced Materials Chemistry Group (GQMAT), Analytical and Physical-Chemistry Department, Federal University of Ceará (UFC), Campus do Pici 12100, CEP 60451-970 Fortaleza-CE, Brazil; E-Mails: antoniocesarhb@hotmail.com (A.C.H.B.); vivisantiago_@hotmail.com (V.R.S.); fael_freire19@yahoo.com.br (R.M.F.); 2 Products Laboratory and Process Technology (LPT), Department of Organic and Inorganic Chemistry, Federal University of Ceará, Fortaleza-CE 12100, Brazil; E-Mail: selma@ufc.br; 3 Physical Department, Santiago University of Chile (USACH), Av. Ecuador 3493, Santiago 9160000, Chile; E-Mail: jcdenardin@gmail.com; 4 Department of Engineering for Innovation, University of Salento, Via Arnesano, Lecce 73100, Italy; E-Mail: giuseppe.mele@unisalento.it; 5 Laboratory of Polymers and Materials Innovation (LPIM), Department of Organic and Inorganic Chemistry, Federal University of Ceará, Ceará 12100, Brazil; E-Mails: cavalcante@gmail.com (I.M.C.); mribeiro@ufc.br (M.E.N.P.R.); nagilaricardo@gmail.com (N.M.P.S.R.); tlemos@dqoi.ufc.br (T.L.G.L.); opessoa@ufc.br (O.D.L.P); 6 Department of Pharmacy, Federal University of Ceará (UFC), Fortaleza-Ceará 12100, Brazil; E-Mail: tamara.ufc@gmail.com; 7 National Nanotechnology Laboratory, Nanoscience Institute-CNR Via Arnesano, Lecce 73100, Italy; E-Mail: luigi.carbone@nano.cnr.it

**Keywords:** drug delivery, cancer therapy, magnetic nanoparticles, oncocalyxone A

## Abstract

This paper describes the investigation and development of a novel magnetic drug delivery nanosystem (labeled as MO-20) for cancer therapy. The drug employed was oncocalyxone A (onco A), which was isolated from *Auxemma oncocalyx*, an endemic Brazilian plant. It has a series of pharmacological properties: antioxidant, cytotoxic, analgesic, anti-inflammatory, antitumor and antiplatelet. Onco A was associated with magnetite nanoparticles in order to obtain magnetic properties. The components of MO-20 were characterized by XRD, FTIR, TGA, TEM and Magnetization curves. The MO-20 presented a size of about 30 nm and globular morphology. In addition, drug releasing experiments were performed, where it was observed the presence of the anomalous transport. The results found in this work showed the potential of onco A for future applications of the MO-20 as a new magnetic drug release nanosystem for cancer treatment.

## 1. Introduction

*Auxemma oncocalyx* Taub belongs to the Boraginaceae family and is an endemic plant of the northeastern Brazilian area, particularly of “caatinga”, a biome exclusive of this region. *A. oncocalyx* is a medicinal plant known as “pau branco” [1–3]. Its stem bark possesses astringent properties used to treat wounds. Previous phytochemical investigation of heartwood extracts of *A. oncocalyx* has resulted in the isolation and characterization of several uncommon meroterpenoid quinones and hydroquinones with a C_16_ backbone [1,3–5]. The major compound isolated from *A. oncocalyx* was oncocalyxone A (onco A), a meroterpenoid quinone, which was obtained in significant amounts.

**Figure 1:**
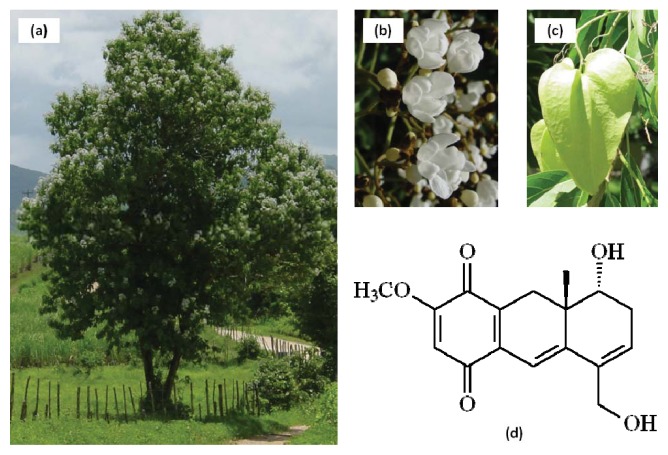
(**a**) An exemplar; (**b**) Flowers; and (**c**) fruit of *Auxemma oncocalyx* in your natural habitat; (**d**) Chemical structure of oncocalyxone A (onco A).

Onco A (Figure 1), *rel*-8α-Hydroxy-5-hydroxymethyl-2-methoxy-8aβ-methyl-7,8,8a,9-tetrahydro-1,4-anthracenedione, a deep red powder, was the first compound isolated from the heartwood extract of *A. oncocalyx*. This secondary metabolite is responsible for the dark color of the heartwood of this plant. Onco A exhibited a series of pharmacological properties, such as cytotoxic, analgesic, anti-inflammatory, antioxidant and causative of inhibition of platelet activation. It also showed differential antitumor activity against the murine tumors Ehrlich carcinoma, sarcoma 180 and L1210 leukemia [2,6]. In general, the active compounds are isolated in small quantities, however, onco A is the major compound produced by *A. oncocalyx* (0.13% from the crude extract). This, when combined with its active properties makes onco A, a suitable compound for drug development.

In cancer therapy, a major difficulty is to destroy tumor cells without harming the normal tissue [7]. A drug that targets tumors, as in other pathological conditions, is desirable since anticancer agents demonstrate nonspecific toxicities that significantly limit their therapeutic potentials [8]. Magnetically-guided particles are regarded to have excellent potential as drug targeting carriers due to their non-invasive character and high targeting efficiency [9]. Through application of an external magnetic field, magnetic drug carriers could be retained to achieve very high concentrations of the chemotherapeutic agent near the target site for a given period of time without any toxic effects to normal surrounding tissue or to the whole body [10]. The factors influencing the release of drugs from magnetic nanoparticles include viscosity of the polymer, the polymer to drug molar ratios, particle size, solubility of the drug, the presence of the magnetic field or additives as well as the incorporation mode of these substances.

Recently, nanoparticles showing a magnetic character, particularly Fe_3_O_4_, have generated a lot of interest in biomedical applications for magnetic resonance imaging, magnetic separation, targeted drug delivery, tissue engineering, cell tracking, bioseparation and magnetic hyperthermia [11,12]. For these applications, the particles must have high magnetic saturation, biocompatibility and interactive surface functionalities [13]. Studies *in vivo* have shown that Fe_3_O_4_ nanoparticles are relatively safe, as they do not accumulate in the vital organs and are rapidly eliminated from the body [14,15]. The presence of a polymeric coating, such as polyethylene glycol (PEG), can furthermore relieve Fe_3_O_4_ citotoxicity, as demonstrated for human fibroblasts [16,17]. In this regard, several experimental approaches have been developed in order to coat iron oxide nanoparticles either during the synthesis procedure (*in situ*) or through post-synthetic treatments. The most common examples of nanoparticle molecular coatings reported in literature are PEG, polyvinyl alcohol (PVA), dextran, alginate and chitosan [18].

In this work, we have reported a new approach to onco A storage and release using Fe_3_O_4_ magnetite nanoparticles. This system was solubilized in the cores of micelles of diblock copolymer, E_114_CL_20_, where E denotes a chain unit derived from ethylene oxide CH_2_CH_2_O, CL a carbonyloxypentamethylene group COO(CH_2_)_5_ derived from ɛ-caprolactone, and the subscripts denote average chain lengths. This copolymer is a biocompatible surfactant, the micelles of which have stealth properties derived from the hydrophilic E_114_ blocks of the corona and good solubilizing capability for hydrophobic agents due the high hydrophobicity of the poly-ɛ-caprolactone blocks of the micellar core.

This copolymer also shows a low critical micellar concentration (see Table 1) with a great potential to be used in aqueous formulations for intravenous administration of hydrophobic drugs [19].

In addition, we also focused on the release study of a promising drug onco A, isolated from a widespread Brazilian native plant, which demonstrated significant effects in cancer therapy [20–22].

## 2. Results and Discussion

**Figure 2:**
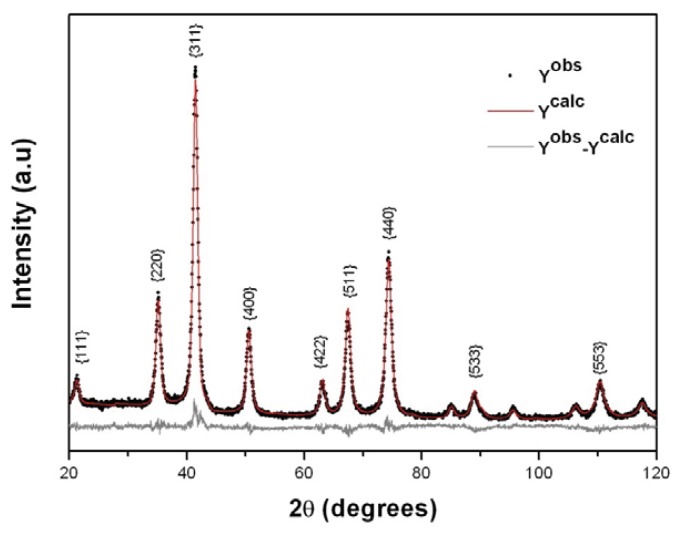
X ray powder diffraction pattern of Fe_3_O_4_.

The Fe_3_O_4_ nanoparticle crystalline structure and size were initially investigated by XRD analysis (Figure 2). The grey lines represent the relative difference between experimental (*Y_Exp_*) and calculated (*Y_Calc_*) intensities obtained by the Rietveld refinement. The diffraction patterns showed that the main reflection peaks at {111}, {220}, {311}, {400}, {422}, {511}, {440}, {533} and {553} can be well indexed with the reference pattern of the Fe_3_O_4_ inverse cubic spinel structure (JCPDS card # 08-4611) with spatial group Fd3M. The crystal size calculated by using the Scherrer’s equation [23] showed a magnetite nanoparticle average size of about 11 nm.

**Figure 3:**
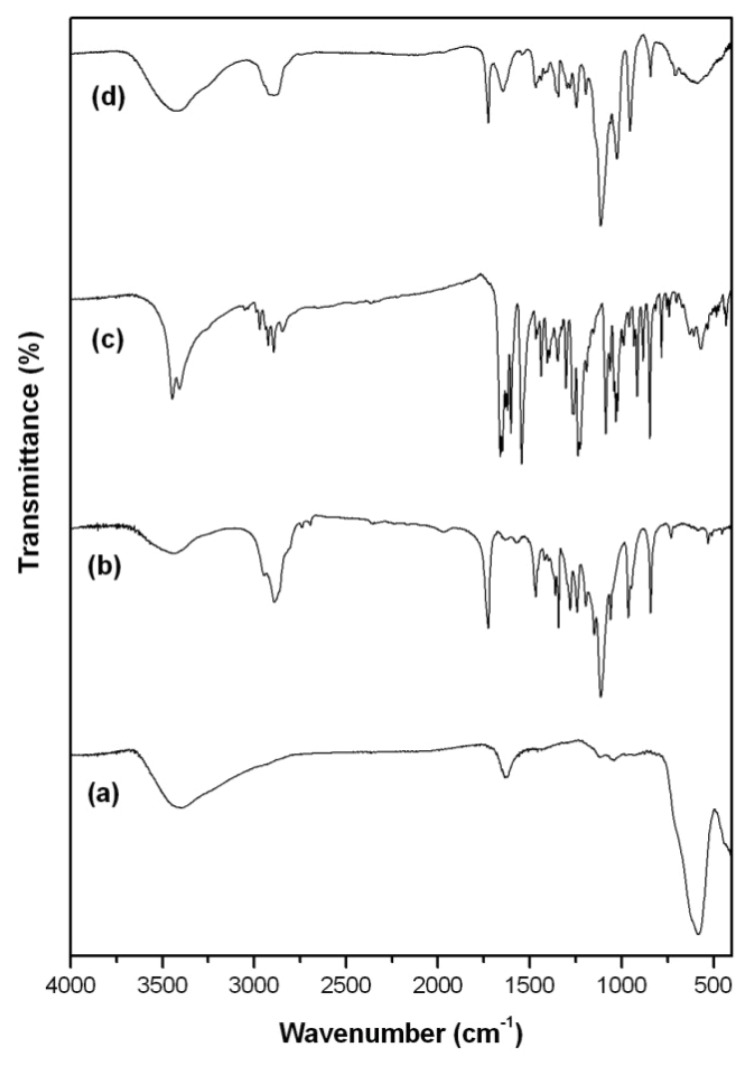
FTIR spectra: (**a**) Fe_3_O_4_; (**b**) Diblock copolymer E_114_Cl_20_; (**c**) Oncocalyxone-A and (**d**) MO-20.

FTIR analysis is one of the most important techniques for a quick and efficient identification of encapsulated chemical molecules [13,24]. In Figure 3a, it was observed characteristic bands of Fe-O around 560–580 cm^−1^ for Fe_3_O_4_ as the main phase of spinel ferrites and corresponds to stretching vibration in tetrahedral site [25,26]. Fe_3_O_4_ has the general molecular formula (Fe^2+^)[Fe^3+^]_2_O_4_^2−^, where the divalent and trivalent cations occupying tetrahedral (Fe^2+^) and octahedral [Fe^3+^] interstitial positions of the fcc lattice are formed by O^2−^ ions [27]. The presence of hydroxyl groups that reside at the Fe_3_O_4_ nanoparticles surface was observed in the broad absorption of O–H stretching at 3411 cm^−1^ for Fe_3_O_4_ and the band at 1637 cm^−1^ is due to angular vibration of O–H. Due to spinel ferrites’ synthesis procedure performed in aqueous environment, the surface materials were covered by hydroxyl groups from water, so that the IR spectrum showed these bands [13]. Figure 3b reports FTIR spectrum for copolymer E_114_Cl_20_ showing characteristic bands respectively at 1114 cm^−1^ associated to aliphatic ether chemical bond (C–O–C), 1730 cm^−1^ due to C=O vibrations present in the ɛ-caprolactone unit and 2881 cm^−1^ associated to aliphatic chains. Figure 3c shows respectively bands of O–H stretching at 3430 cm^−1^, of C–H stretching at 2930 cm^−1^, of C=O stretching at 1660 cm^−1^ and of aliphatic ether C–O–C as principal band present in Onco A (see Figure 1). In Figure 3d, bands associated with the copolymer (1114 cm^−1^, 1730 and 2881 cm^−1^) and the drug (3430 cm^−1^, 2930 cm^−1^ and 1660 cm^−1^) were observed. However, it was also observed a band around 560–580 cm^−1^ characteristic of Fe-O vibration mode. These results suggested the encapsulation of the magnetite in the nanoparticles of the polymer-drug to obtain the nanosystem (MO-20).

**Figure 4:**
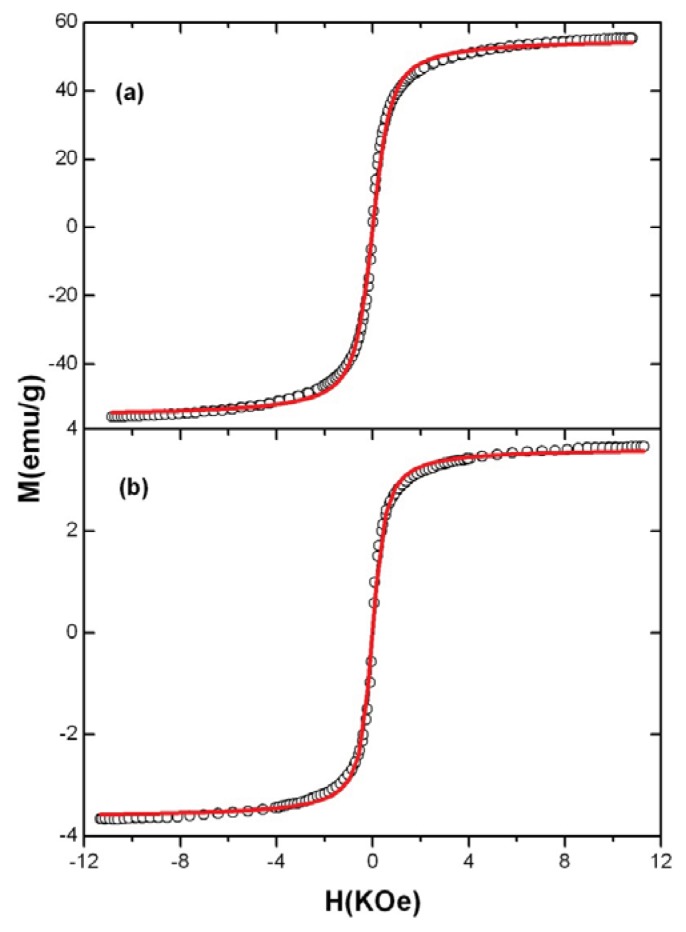
Magnetization curves of (**a**) Fe_3_O_4_ and (**b**) MO-20. The solid lines correspond to the fitting with the Langevin equation (see text).

VSM was performed to investigate the magnetic properties of Fe_3_O_4_ and MO-20 at room temperature. In Figure 4a, the hysteresis loops that are characteristic of superparamagnetic behavior can be observed for Fe_3_O_4_ nanoparticles. There is no hysteresis in the magnetization curve with both remanence and coercivity being zero, indicating that these magnetic nanoparticles are superparamagnetic. This feature is an important property needed for magnetic targeting carriers, because capillary blockage by aggregations formed by residue magnetism after removal of the applied field will be avoided [10]. The saturation magnetization of MO-20 (Figure 4b) is found to be 3.65 emu/g. This value is significatively lower than for magnetite nanoparticles (55 emu/g) reasonably due to the three coating layers of oleic acid, onco A and copolymer. Hu *et al.* [10] observed an 86% decrease in saturation magnetization with the addition of organic compounds on the surface of magnetite. Barreto *et al.* [13] observed a 94% decrease in saturation magnetization after encapsulation with quercetin and triblock copolymer. This feature renders the nanoparticles promising candidates for highly efficient magnetic manipulation when used as drug delivery carriers [28].

**Figure 5:**
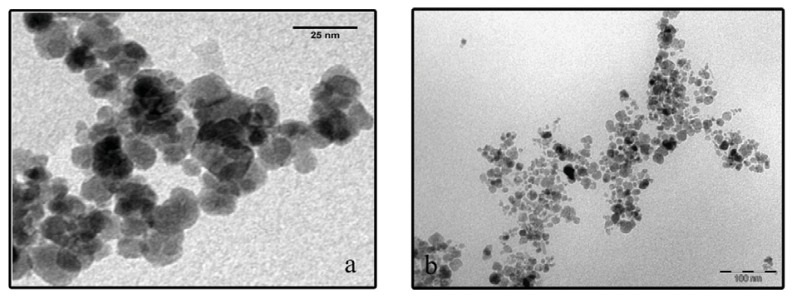
TEM images of (**a**) Fe_3_O_4_ and (**b**) MO-20.

The room temperature hysteresis curves (Figure 4a,b) *M*(*H*) of these samples can be well described by a Langevin function, *M/M*_0_*=* coth (μ*H*/*k*_B_) − *k*_B_*T*/μ*H* with μ the magnetic moment, *H* the external magnetic field, *T* the temperature, and *k*_B_ the Boltzmann constant. The particle size can be inferred from the Langevin function adjusting the parameter *a* = μ/*k*_B_, which is related to the particle diameter *d* as *a* = 4π(*d*/2)^3^M_0_/3*k*_B_. Thus, by using this fitting with parameters *a* of 1 and 1.4 gives us average diameters respectively of 11.5 nm for Fe_3_O_4_ and 29.8 nm for MO-20. These results agree with the magnetite size determined by XRD and TEM (see Figure 5), respectively. Similar outcomes were demonstrated by Barreto *et al.* [13] upon studying the hysteresis loop of quercetin and the triblock copolymer E_137_S_18_E_137_-coated magnetite nanoparticles.

The Fe_3_O_4_ nanoparticles and MO-20 nanosystem morphology were furthermore investigated by TEM analysis, as shown in Figure 5. Size polydisperse Fe_3_O_4_ nanoparticles maintain a round-shaped profile with an average crystallite size of 13 nm typically agglomerated according to flower-like nano-architectures due to interparticle magneto-dipole interactions. In the case of MO-20 nanosystem, the particles showed less tendency to aggregation, rationally due to organic coating-promoted reduction of Fe_3_O_4_ surface energy and dipolar attraction, with a substantial increase of the overall particle size up to 30 ± 2 nm. These results accord with sizes determined by XRD and VSM analyses (Figures 2 and 4, respectively). It was also observed the decrease of the agglomeration by TEM after modifications in the surface of magnetic nanoparticles [29,30].

**Figure 6:**
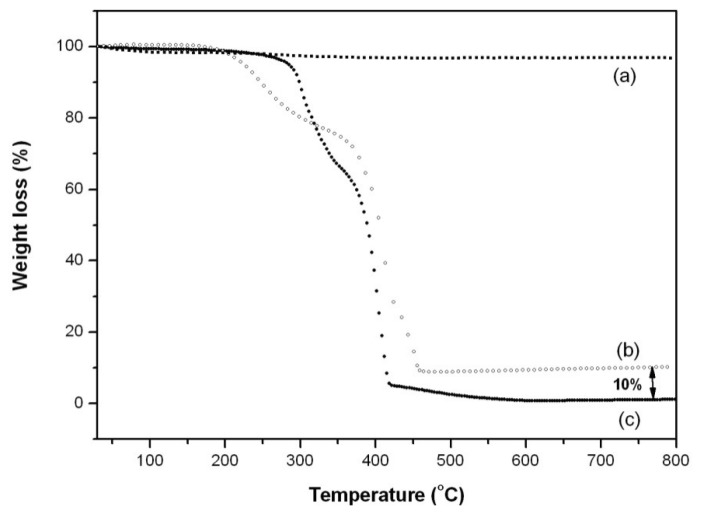
Weight loss by thermogravimetric analysis (**a**) Fe_3_O_4_; (**b**) MO-20 and (**c**) copolymer.

To investigate thermal behavior and determine the amount of magnetic nanoparticles present in MO-20, TGA were performed and illustrated in Figure 6. The magnetite amounts from samples can be estimated from the residual mass percentages. Magnetic nanoparticles powder showed practically no weight loss in the temperature range 25 to 800 °C due to their high thermal stability (Figure 6a). On the other hand TGA data for MO-20 nanosystem demonstrated that the first weight loss occurs at about 200 °C (Figure 6b), which is 100 °C lower than for the pure copolymer (Figure 6c). This shift in the temperature might be ascribed to the presence of oleic acid and onco A multilayers in MO-20. In both cases a second degradation starts around 325 °C indicative of a mass loss of 25 and 32 wt% for MO-20 and the copolymer, respectively. As illustrated in Figure 6b the remaining metal nanoparticle (magnetite) content is almost 10 wt% in MO-20, whereas copolymer thermograms showed a complete weight loss over the temperature range 25 to 800 °C. Ilgin *et al.* [31] studying magnetic composites based on hyaluronic acid hydrogel observed 20% nanoparticle weight content by TGA analysis.

**Figure 7:**
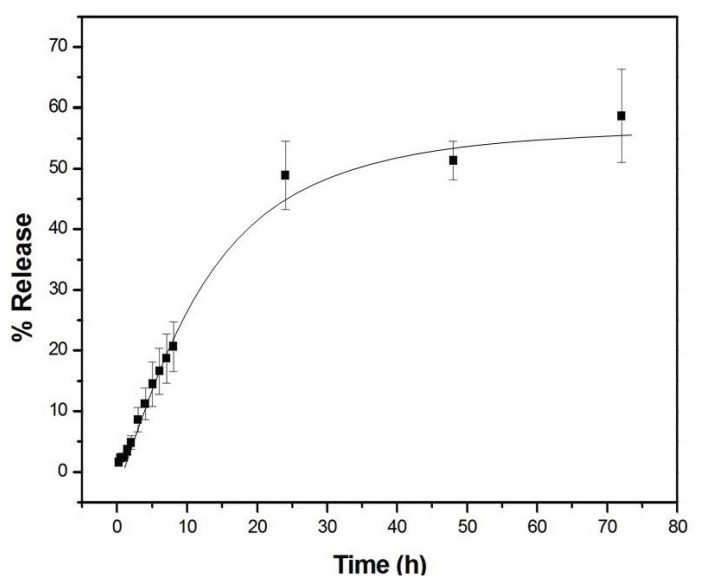
Release profile of onco A from MO-20 by UV-Vis method.

The release study was carried out in triplicate and the solid line is for eye guidance (Figure 7). In the first 10 h, there was initial rapid burst release. This fact is normally attributed to the fraction of onco A adsorbed on the surface of the diblock copolymer [13,24]. The cumulative release of onco A as a diffusion controlled process under a physiological condition (pH = 7.4 and incubated at 37 °C) shows a gradual increase and achieved its maximum value at about 60% after 72 h.

In order to figure out the mechanism of drug releasing from the matrix system, the experimental data were fitted utilizing Higuchi [32] as well as Korsmeyer-Peppas [33,34] models, both based on an exponential relationship of drug release as a function of time. The kinetics parameters and correlation coefficient (*r*^2^) obtained from the analysis are shown in Table 2. Both models were well fitted, with *r*^2^ values varying in the range 0.9709–0.9831. Upon correlating the Higuchi [32] and Korsmeyer-Peppas [33] models, it was observed that the latter presents higher values of *r*^2^, this may indicate the existence of another releasing contribution in addition to a Fickian diffusion. Whether the Higuchi [32] model assumes that uniquely a Fickian diffusion governs the release process [32,35].

The Korsmeyer-Peppas model [33] uses the parameter *n* to characterize the mechanism of drug release. Depending on the *n* values, different types of drug delivery regimes can be identified [36]. Specifically, if *n* is in the range 0.4–0.5, the release merely follows a Fickian diffusion, whereas if *n* = 1, a case-II transport occurs leading to a zero-order release. Finally, for values in the range 0.5–1.0 a so-called anomalous transport takes place, whereby Fickian diffusion and case-II transport simultaneously dominate. The analysis of the release curve (Figure 7) gives *n* = 0.57 ± 0.02, indicating the presence of an anomalous transport. However, *n* value is very close to 0.5, thus indicating that the predominant drug release mechanism is due to Fickian diffusion.

## 3. Material and Methods

### 3.1. Materials

The chemical reagents for this work are FeCl_3_·6H_2_O (pure granulated 99%), FeSO_4_·7H_2_O (pure granulated 99%) and 30% ammonia solution. Copolymer E_114_Cl_20_ was prepared by sequential oxyanionic polymerization using a vacuum line ampoule technique based on experimental procedures described elsewhere [37,38]. Values of the molecular characteristics are listed in Table 1. Chloroform, ethyl acetate and oleic acid were used without further purification.

The extraction and isolation of onco A, from the heartwood of *A. oncocalyx*, was carried out as described earlier [1,4] and its structural identity was confirmed by thin layer chromatography (comparison with authentic sample) and ^1^H- and ^13^C-NMR spectral analysis (Table 3).

### 3.2. Oleic Acid-Coated Fe_3_O_4_ (OA-Fe_3_O_4_)

The synthesis of magnetite particles has been already described in previous studies [13,39]. Briefly in a co-precipitation processing route, two aqueous solutions of metallic salts containing Fe^2+^/Fe^3+^ were mixed in Milli-Q water in the molar ratio of 1:2 to form a Fe_3_O_4_ spinel phase. The aqueous mixture was heated at 80 °C and then, under vigorous stirring, a 30% wt NH_4_OH solution added up to pH 10 with the formation of a black precipitate. The precipitate was washed several times with Milli-Q water until the residual solution became neutral. The chemical reaction of Fe_3_O_4_ formation may be written as follows

(1) $${\text{Fe}}^{2+}+2{\text{Fe}}^{3+}+8{\text{OH}}^{-}\to {\text{Fe}}_{3}{\text{O}}_{4}+4{\text{H}}_{2}\text{O}$$

Subsequently, oleic acid was added in a significant excess amount to the system right after the co-precipitation had started, which resulted in the chemisorption of the acid on the magnetite surface, as confirmed by the OA-Fe_3_O_4_ solubility in organic solvents. This was followed by a washing process using ethanol with magnetic decantation to remove the no-dispersed particles.

### 3.3. Synthesis of OA-Fe_3_O_4_ Coated with Onco A-E_114_Cl_20_ (MO-20)

**Figure 8:**
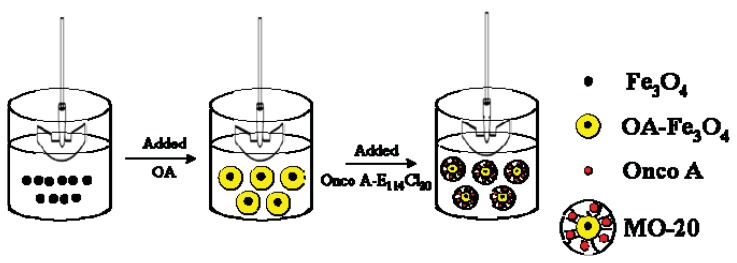
Schematic illustration of the reaction to obtain MO-20 nanosystem.

The magnetic drug delivery nanosystem was prepared by the emulsion-coacervation method [10,13] followed by a polymer coating procedure. In details, a sample of OA-Fe_3_O_4_ (30 mg) was initially dispersed in 100 mL of chloroform, onco A (30 mg) dissolved in ethyl acetate and 150 mg of the diblock copolymer in chloroform. Upon using a mechanical stirrer (1000 rpm) OA-Fe_3_O_4_ and drug solutions were mixed and then the copolymer solution added to the mixture. Stirring was continued at room temperature for 1 h. Afterwards, the solvent was extracted and the as-obtained solid characterized and labeled as MO-20. Schematic illustration of each single experimental step is shown in Figure 8.

### 3.4. Oncocalyxone Releasing from Copolymer E_114_Cl_20_

MO-20 samples were diluted in phosphate-buffered saline solution (PBS, pH = 7.4) and then transferred into dialysis membrane. The membrane was placed into the same buffered solution (25 mL). The release study was performed at 37 ± 0.5 °C. At pre-determined time intervals, 3 mL of the aqueous solution were withdrawn and filtered through Millipore Millex filters (0.45 μm) and replenished with 3 mL of fresh buffer solution. The amount of released drug was measured through absorbance using UV spectrophotometer at 375 nm for onco A. The initial drug concentration in the dialysis membrane was 40 μg/mL. In the assessment of drug release behavior, the cumulative amount of released drug was calculated and the percentages of onco A released from the copolymer were plotted against time. A U-2000 Spectrophotometer by Hitachi was used in the analysis. All release experiments were performed in triplicate and the mean values employed to build release profiles. The obtained drug release data were analyzed according to the Higuchi [32] and Korsmeyer-Peppas [33] equations.

### 3.5. Characterization

^1^H (500 MHz) and ^13^C (125 MHz) and 2D NMR experiments were performed on a Bruker Avance DRX-500 spectrometer equipped with a 5 mm inverse detection z-gradient probe. ^1^H and ^13^C NMR spectra were measured at 27 °C using dimethylsulphoxide as solvent. Column chromatography was run using silica gel 60 (70–230 mesh, Vetec and 230–400 mesh, Merck, Darmstadt-Germany), and TLC performed on precoated silica gel polyester sheets (Kieselgel 60 F_254_, 0.20 mm, Merck, Darmstadt-Germany) by detection with a spraying reagent of vanillin/perchloric acid/ethanol solution followed by heating at 100 °C.

The X-ray diffraction (XRD) analysis was performed in a X-ray powder diffractometer Xpert Pro MPD (Panalytical) using Bragg-Brentano geometry in the range of 20°–120° with a rate of 1°min^−1^. Co Kα radiation (λ = 1.7889 Å) was used and the tube operated at 40 kV and 30 mA. The phase identification analysis was made by comparing obtained powder diffractograms with standard patterns from International Centre for Diffraction Data (ICDD). For the magnetic nanoparticles, the experimental patterns were numerically fitted with the Rietveld algorithm in a procedure to better identify and quantify crystallographic phases.

Fourier transform infrared spectroscopy (FTIR) measurements were performed using a Perkin Elmer 2000 spectrophotometer in the 400 to 4000 cm^−1^ range. The samples were previously dried and grounded to powder and pressed (10 μg of sample to 100 mg of KBr) in disk format for measurements.

The magnetization measurements were performed at room temperature with a home-made vibrating sample magnetometer (VSM). The VSM has previously been calibrated using a pure Ni wire, and after measuring the mass of each sample the magnetization was given in emu/g.

The thermal stability of the materials was done by Mettler Toledo thermogravimetric analysis/simultaneous differential thermal analysis (TGA/SDTA) 851^e^ machine. The analyses were performed under constant flow nitrogen atmospheres (50 cm^3^/min), with heating rate of 10 °C/min, sample mass of 10 mg and temperature programs in the range 25–800 °C.

Low-magnification Transmission Electron Microscopy (TEM) analysis was performed on a Jeol JEM-1011 electron microscope operating at 100 kV, equipped with a CCD camera ORIUS 831 from Gatan. TEM samples were prepared by drop-casting dilute nanocrystal solutions onto carbon coated copper grids. Afterwards, the deposited samples were allowed to complete dry at 60 °C for one night before examination.

## 4. Conclusions

The results demonstrated that the magnetic drug delivery nanosystem (MO-20) was obtained with association of an inorganic component (magnetite nanoparticles), a monounsaturated fatty acid found naturally in many plants and animals (oleic acid), a synthetic diblock copolymer (E) and a drug isolated from a Brazilian native plant (Onco A). This natural drug was used because it exhibited a series of pharmacological properties as antitumor activity, while Fe_3_O_4_ was used to obtain a system with magnetic properties. The nanosystem size was confirmed by TEM, XRD and hysteresis curve (Langevin function). For MO-20 nanosystem, it was found a size of approximately 30 nm due to the copolymer coverage. Mathematical models were furthermore employed to distinguish the mechanism of drug releasing, whereby the best correspondence with the Korsmeyer-Peppas [33] model, suggests the presence of an anomalous transport (Fickian diffusion and case-II transport), however with a predominant contribution of Fickian diffusion. Changes in the system composition could adequate an ideal release time or change the release mechanism. These results indicate the great potential for future applications of the MO-20 as a new magnetic drug release nanosystem for cancer therapy.

## Figures and Tables

**Table 1:** Molecular characteristics of E_114_CL_20_ copolymer.

Copolymer	*M*n (g·mol^−1^)	*w*_E_	*w*_h_	*M*w/*M*n	cmc (g·dm^−3^)
E_114_CL_20_	7328	0.689	0.311	1.36	0.0089

*w*_h_ = weight fraction of the hydrophobic (CL_20_) block of the copolymer; *w*_E_ = weight fraction of the hydrophilic (E_114_) block of the copolymer; cmc = critical micelle concentration in aqueous solution, temperature 37 °C.

**Table 2:** Mathematical models and parameters used to describe drug release curve from MO-20 nanosystem.

Model	Equation	Parameters		*r*^2^
Higuchi	$\frac{M_{t}}{M_{\infty }}=K_{\text{H}}\sqrt{t}$	*K*_H_		0.9709
		6.8541 ± 0.24		
Korsmeyer-Peppas	$\frac{M_{t}}{M_{\infty }}=Kt^{\text{n}}$	*K*	*n*	0.9831
		5.3243 ± 0.54	0.5717 ± 0.02	

*M*, absolute amount of drug at time *t; M*_∞_, total amount of drug released at infinite time; *K_H_*, higuchi release constant; *K*, kinetics constant; *n*, diffusional exponent.

**Table 3:** ^1^H and ^13^C NMR spectral data for Onco A.

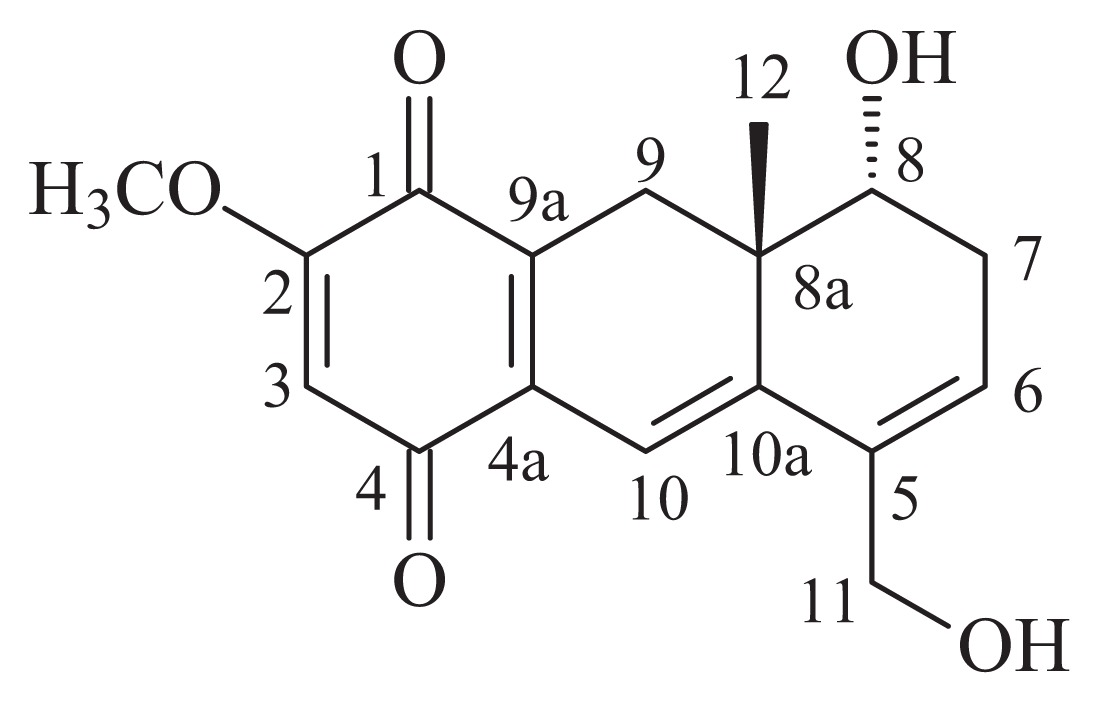		

Position	δ_C_ (ppm)	δ_H_ (ppm)
1	180.7	–
2	159.3	–
3	105.9	6.00 (s)
4	185.5	–
4a	134.1	–
5	135.0	–
6	127.9	6.03 (br s)
7	31.4	2.60 (d, *J* = 18.6 Hz)
8	69.6	2.37 (dd, *J* = 18.6 Hz)
8a	38.8	3.56 (br s)
9	28.7	2.93 (d, *J* = 18.2 Hz)/2.34 (d, *J* = 18.2 Hz)
9a	132.5	–
10	111.3	6.49 (s)
10a	146.2	–
11	61.1	4.17 (br s)
12	20.8	0.74 (s)
CH_3_O	56.2	3.78 (s)
HO-8	-	4.93 (d, *J* = 3.7 Hz)
HO-11	-	4.87 (br s)
